# Philosophy leading the way: An interdisciplinary approach to study communication of severe diagnoses

**DOI:** 10.1371/journal.pone.0305937

**Published:** 2024-07-22

**Authors:** Monica Consolandi

**Affiliations:** 1 Fondazione Bruno Kessler, Center for Digital Health and Well Being, Unit of Intelligent Digital Agents, Trento, Italy; 2 Consultant, Pontifical Academy for Life, Vatican City, Rome, Italy; Pontificia Universidad Catolica de Chile (PUC) / Universidad de Valladolid (UVa), SPAIN

## Abstract

This paper explores a brand-new interdisciplinary approach applied to an enduring problem: the communication of severe diagnoses. The moment when physicians explain the diagnosis to patients and their relatives is sensitive, particularly for a disease that is rarely diagnosed early. The first part of the article is dedicated to the context of this delicate doctor-patient interaction. With this framework in mind, the paper delves into the innovative interdisciplinary methodology developed in the pilot study Communi.CARE, conducted in a hospital in Northern Italy, which focuses on the diagnosis of pancreatic ductal adenocarcinoma (PDAC). SARS-CoV-2 impact on the study development is highlighted. The study aims to explore the topic by combining different areas of expertise, including medicine, philosophy, sociology, and psychology. The contribution of philosophy is here presented as essential: it has a leading role in the conception of the study, its development, and the elaboration of results. It is shown throughout the study, from methodology to the analysis of results. Strengths and weaknesses of the methodology are discussed. In conclusion, further philosophical considerations on effective and ethical communication in this delicate context are recommended.

## 1. Introduction

### 1.1 Significance of effective and trustworthy communication in conveying a diagnosis

Conveying diagnoses is one of the most difficult tasks that a physician has, and one of the most sensitive moments to live for patients and their relatives. Determining a patient’s health condition requires many steps; thus, the precise moment when the information is delivered to the patient and its modalities may vary depending on numerous factors. For example, it may happen after the patient has received clinical results; unexpectedly, as incidental finding; or, amidst the examination of symptoms that seemed to lead somewhere else. Due to its multiform nature, a diagnosis takes place in different settings: it could be the physician’s office as far as the hospital hallway; nowadays, it could also be indirect and happen in a virtual place such as the section of reports of the electronic health record accessible to patients. A specific diagnosis may be predictable, this also calling for different strategies and approaches.

Diagnosis does not take place in a single moment: it is instead a collection of information that gives shape to the overall picture, a process that leads to a name and a definition. Nonetheless, there is a specific point in time when patients (and eventually their families or companions) become aware of their condition. This sensitive occasion is both the end point of clinical investigations and the starting point of the therapeutic pathway, but also an impacting episode in the patient’s story. It is a turning point that originates from communication.

At the origin of the innovative study protocol that is the main subject of this article, there is the strong conviction that the quality of communication should be pursued and always safeguarded: not only it strengthens a positive relationship between the speakers, but it is helpful in creating a solid therapeutic alliance between the patient and the physician [1–4]. Doctor-patient relationship is the core of the therapeutic journey and highly impacts its outcomes, such as the quality of care, patient’s safety, autonomy [5, 6], adherence, and compliance [7–11]. It has already been acknowledged to be a *clinical* tool [12–15]. It is sometimes even legally considered “treatment time” (as it is in Italy: see [16]) and knowing how to communicate properly is considered part of the doctor’s responsibility [17–19].This is especially true in the delicate context of conveying a diagnosis, where patients “submit themselves to be determined in their future condition by the one they consult” (see [20], p. 28).

Due to its relevance in patients’ life, the moment of diagnosis deserves a dedicated setting and a reasonable duration; unfortunately, it is not always possible to guarantee the proper modalities in the complicated hospital setup: lack of time and places to carry it out are among the causes of diagnoses that are conveyed in undesirable settings. Nonetheless, aware communicators can preserve the value of interaction even though circumstances are adverse. Studies show that even though the conversations with the doctors were brief, patients reported perceiving longer durations when the conversation was satisfactory for them [21, 22]. This reinforces the notion that well-conducted communication is of utmost importance, not only in terms of patients’ perception but also as a starting point (or transitional phase) within the therapeutic journey [23].

Effective physician-patient interaction means surely conveying clear information, but also being compassion and empathic with a human being that is experiencing a turning point in life. Doctors with communication competence are more likely to be understood by patients and are more likely to establish a cooperative relationship with them [24–26]. In other words, a communication of quality cannot be simplified to a to-do-list that the doctor checks during the interview, nor should reduce the patient to the diagnosed disease; instead, it calls for an in-depth vision of the phenomenon and the advancement of specific skills.

### 1.2 The case of severe diagnoses

Given that a quality communication is always essential, it is worth it to place heightened emphasis to the way we speak in thorny circumstances; this happens, for example, when we are engaging with vulnerable subjects, conveying important information, and delving into sensitive topics [27–29].

All the above conditions are present in the context of severe diagnoses. Indeed, the patient is always a vulnerable subject; particularly when the condition of a severe diagnosis is met. Serious medical verdicts inevitably call for existential changes that should be acknowledged within the dialogue between clinicians and their patients. Vital information is conveyed by the physician about delicate issues, such as the description of the disease, suggested treatment, and how it affects patient’s life. At this moment, their conversation is not only about life, but also calls for the inevitability of death and its proximity. Patients are people, meaning that they do have a story and a present. They have roots, personal beliefs, come from different educational backgrounds, professional careers, and variety of experiences. They have families, friends, hobbies, concerns, and they look at the world with their unique lenses. The impact of a grave diagnosis is not predictable; but it is not hard to envision the collision on a human life.

The diagnosis of pancreatic ductal adenocarcinoma (PDAC) is a solid example. In fact, PDAC is an especially aggressive form of cancer, characterized by a bleak outlook resulting from diagnoses often made in advanced stages and limited responses to available therapies. These combined factors contribute to a high mortality rate, as the 5-year survival rate stands at a mere 11% [30]. Therefore, we are dealing with a diagnosis that frequently comes as a surprise and occurs abruptly. Individuals who receive this diagnosis are embarking on a complex journey involving acceptance, treatment, and hardship. Delicacy and clarity of information are cornerstones in the communication of such a complex diagnosis.

## 2. The study protocol Communi.CARE

Within this framework, Communi.CARE (Communication and Patient Engagement at Diagnosis of Pancreatic Cancer) originated. It is a pilot study (i.e., first in its genre and with a relatively small sample of enrolled participants) that addresses the topic of effective communication in the context of the diagnosis of PDAC. It aims to find possible correlation between patients’ understanding, engagement, and compliance. It has been conducted in San Raffaele Hospital, a large hospital in the North of Italy; its Pancreas Translational and Clinical Center, structured in Clinical Units that mirrored the hospital departments but with a strong multidisciplinary approach to the disease, offers a solid framework for our research. Besides, the Department of Pancreatic Surgery is one of the excellences in Europe for its number of operations and low mortality (see the webpage https://www.gsdinternational.com/hospitals/ospedale-san-raffaele/pancreatic-surgery, accessed on August 29, 2023). The full protocol can be consulted in open access [31] and in ClinicalTrials.Gov (NCT04257955). Results can be read in [32].

Communi.CARE originates with the idea of studying the communication of PDAC diagnosis with a humanistic-leading approach. The desired outcome is a comprehension of this sensitive moment that includes all the elements at stake: the subjects (the patients and their families, the physicians, and the disease), the technical information to be conveyed, and the emotions implied due to the life-changing nature of the diagnosis. The final outcomes may result in the development of guidelines or directions for clinicians to manage the conveying of this severe diagnosis to the best of the possibilities, all the above-mentioned elements considered.

### 2.1 An interdisciplinary project: Advantages and hindrances

The conception of this study is strongly characterized by the idea that the interdisciplinary approach is of profound utility, especially when applied to real-case scenarios. The article considers as a reference to distinguish the definitions of *multidisciplinary*, *interdisciplinary*, and *transdisciplinary* [33]. Thus, the research here described is considered interdisciplinary due to the close cooperation of the different fields involved. This research attitude is underscored by the involvement of various disciplines converging to investigate a shared subject and guided by philosophy to reach results and discuss about them.

Even though the need for research about this topic was high and shared among clinicians who deal every day with this severe disease, the journey to develop the design of the study has not been obvious. Exactly because of its interdisciplinary roots, it required time for all the actors involved to find each other and to create the teamwork: the dialogue between philosophers and clinicians (the experts who collaborated at this project) is not yet common and for sure has not dedicated spaces. The challenge was then to merge different methods to organize all the fields of expertise involved and thus succeed in the final interdisciplinary discussion. Perspectives from both *hard* and *soft* sciences had to be combined with the idea in mind to overcome this distinction and cooperate on a shared important topic as equals. On this topic, see the famous work of Charles Percy Snow [34]. Also, Oliver Sacks in his autobiographical book romantically reminds to the reader the times when humanities and sciences were not sharply distinguished but worth of the same consideration; as a testament to that, chemists and scientists of the 19^th^ century wrote about chemical components as well as poems in Chapter XI, *Humphry Davy*: *a chemical poet* [35].

Who have already performed interdisciplinary research know that it is not always easy for researchers to understand each other when coming from different fields. It happens frequently to use the same term with different meaning; not to fully grasp the reciprocal technicalities; and to experience difficulties in combining different methods. A continuous dialogue between the actors involved and the common intent to pursue the project’s goal of a comprehensive image of the topic as exhaustive as possible allowed to overcome and solve this kind of hurdle.

The first tangible result of the effort is the study protocol per se as it was structured to be presented to the Ethical Committee for Clinical Experimentation (in Italy, it is the equivalent of the American IRB, Institutional Review Board). Even though this is a mandatory passage when research involves human beings, it has not been obvious how to present the study based on forms required from the hospital. In fact (especially in Italy), templates are often standardized based on clinical trials, thus using quantitative language that is not always suitable for interdisciplinary research. We thus had to *translate* the description of our protocol to fit a numerical standard and, of course, this was not easy as our primary concern in this phase was not to distort our perspective. We ended up with an adequate version that not only was understandable and acceptable for the Ethical Committee, but also represented the first demonstration that collaboration between markedly dissimilar fields of expertise can yield positive results.

Despite the harshness, the effort was worth it to pursue a collaboration that not only is based on scientific premises, but also integrates the human perspective in its approach to patients. The importance of structuring research this way is even more determinant if we consider that it may hopefully reflect on the way of structuring care and medicine itself.

### 2.2 SARS-CoV-2 impact: Changes and limitations

Since the beginning of the study took place in 2020, when SARS-CoV-2 spread all around the world, data collection was slower than expected: it is notable that SARS-CoV2 determined a significant drop of complex diagnoses that require many exams to be identified. In May 2020, it is estimated that there was already a backlog of 4 million tumor screenings to catch up with previous years (see https://www.univadis.it/viewarticle/screening-rimandati-per-covid-quasi-4-mln-test-per-mettersi-in-pari-720071, accessed on August 29, 2023; for a complete report about the Italian situation, see https://www.osservatorionazionalescreening.it/sites/default/files/allegati/Rapporto%20ripartenza-12_20.pdf, accessed on August 29, 2023). After only 1 year, Sanità24 reported 100 million screenings and 1 million fewer cancer diagnoses in Europe (see https://www.sanita24.ilsole24ore.com/art/europa-e-mondo/2021-06-17/covid-europa-100-milioni-screening-e-1-milione-diagnosi-cancro-meno-154636.php?uuid=AE8v10Q, accessed on August 29, 2023).

This unfortunate effect of the virus is one of the collateral consequences on patients with (maybe not yet diagnosed) diseases that did not fall within the category of Covid-19.

In our study protocol, the impact of SARS-CoV-2 is evident in two main areas: data collection and doctor-patient communication.

Italian Decrees mandated that only essential healthcare personnel could enter hospitals (see DL 23 febbraio 2020 n.6; DL 25 marzo 2020 n.13; L. 5 marzo 2020), and most of research projects had to be suspended. Luckily, we managed not to interrupt our data collection, instead assigning the task to audio-record the visits and obtain informed consents from patients to the involved physicians. Nonetheless, body language and context details went missed, since no one external to the conversation could take notes. The bright side of this change is that there was no external observer possibly influencing the scene. Furthermore, the interviews with patients took place on the phone: even though patients were most of times in a comfortable environment (e.g., their home, their car), sometimes they agreed to be interviewed while they were waiting for follow-up visits or exams and thus anxious and in a hurry. Nonetheless, some of them openly thanked me to help them spending time and get distracted during the wait.

It was also difficult to establish a connection with them: it is considerably arduous in scenarios where in-person communication is absent, and all dialogue unfolds in a virtual environment.

Since communication is the focus of the study protocol we are describing, it is worth to notice that we involuntarily happened to observe it in a unique scenario–or, at least, in a rare scenario. SARS-CoV-2 directly modified doctor-patient communication: the extraordinary framework had undeniable repercussions both on the practical and emotional side of care. During the pandemic period, the mood of many reflected the general atmosphere of tension. Physicians and patients had to wear masks and their conversation was affected by this requirement, since it causes lack of facial expressions and thus erases information conveyed through body language. Due to the emergency, it was not recommendable to admit anyone other than the patient themselves to the visit; however, the physicians involved in this study showed their empathy deciding to allow one person (a relative or a friend) to be in the office with them.

## 3. The role of philosophy

Philosophy plays a prominent role in this interdisciplinary study protocol. It is possible to detect its contribution in each step: conception, methodology, and elaboration of results. Each passage is examined below and the role of philosophy as the guiding field of the overall research is shown.

### 3.1 Philosophy in theory

The study framework of Communi.CARE is philosophical: from the very beginning, its development started from the willingness to study the interaction between physicians and patients within a philosophical framework. Not coincidentally, the purpose of the study was shared by clinicians: this confirmed that there was a need for philosophical contribution, as it was perceived as relevant by the key actors. In fact, later in the study we would have found out that patients were also very happy to contribute with their voices to a study that met their needs. It was undoubtedly satisfying to verify that the thoughts at the origin of the study here described were accurate.

The conception took root in the principle that sees communication and trust as strongly intertwined [36–38]. Figuratively speaking, the presence of a disease induces the body to speak through symptoms. Since most of us are experts neither in this language nor in giving answers to a sick body seeking relief, we need someone to do that for us. “We put our bodily safety into the hands of […] doctors, with scarcely any sense of recklessness,” writes Baier (see [39], p. 234). That is why “trust is important, but it is also dangerous” [40]. Even though we must entrust ourselves to physicians, blind trust in doctors is not typical of today’s medicine. Especially with the advent of the Internet, the phenomenon of self-diagnosis has increased [40], and many patients think themselves capable of interpreting symptoms on their own. Effective communication is thus all the more important for building an effective therapeutic relationship based on trust to achieve the expected health outcomes.

“Trust, the phenomenon we are so familiar with that we scarcely notice its presence and its variety, is shown by us and responded to by us not only with intimates but with strangers”, says Baier [39]; such is the case with the doctor–patient relationship. When people ask for medical advice, they become patients; in Sokolowski’s words, they “submit themselves to be determined in their future condition by the one they consult” (see [20], p. 28), i.e., the physician. This is the beginning of the relationship and the time that the doctor–patient interaction begins. Patients rely on physicians to restore their health. They trust them to be able to understand the meanings of their symptoms and to cure their illnesses in the best way possible. Along with Clark, “as patients […] we trust physicians to keep our health concerns first and foremost” (see [3], p. 11). Patients trust physicians to the point of getting naked in front of them (what Sokolowski [20] call “the phenomenon of nakedness,”, p. 28)—both literally, by getting undressed, and metaphorically, by sharing intimate information and relying almost completely on their expertise.

In [41] a meta-analysis is conducted to demonstrate that, despite smaller correlations “with health behaviors, quality of life and symptom severity” (p. 1), there is a correlation between patients’ trust in healthcare professionals and the patients’ satisfaction (on this topic, see also [42]). Rhodes goes beyond noting this positive correlation, claiming that “the first duty of medical ethics is to seek trust and be deserving of it” ([43], p. 51), and at the same time remarking on the strong link between trust and trustworthiness that we already find in [44, 45]. Rhodes asserts that this duty is “the source from which all of the other more specific duties are derived” ([43], p. 53), considering trust and trustworthiness to be the basis of good medical practice and a strong doctor–patient relationship.

As patients, we need to trust physicians to accept their interpretation of what our body is telling us in situations in which the “outcomes are unknown” [46], i.e., in cases of disease. Physicians’ trust in patients, on the other hand, contributes to increasing physicians’ well-being and satisfaction, thus creating positive feedback between them and their patients. This mutual form of trust enhances patients’ trust in their physicians and vice versa [1, 2].

The doctor–patient relationship is “a keystone of care” and is developed and manifested mostly during medical interviews, which are “the major medium of health care” and thus the privileged venue for fostering trust. It may be surprising to note that even though most of the medical interview is “spent in discussion” [47], during their entire course of treatment patients rarely have the chance to relate with the experts to whom they are entrusting their health. Throughout the series of medical exams, pharmacological therapy, and surgical procedures, there are few occasions when physicians and their patients meet, speak to each other, and communicate about the patients’ health and decisions to be taken. This implies that it is even more important for both the parties of the relationship—the doctor and the patient—to understand and be able to manage the dynamics of language and communication to orient within the medical encounter. The moment when the physician conveys the diagnosis here described is a great example.

Trust is “just one way in which a trust relationship may be begun” ([48], p. 10). Such a relationship may instead begin with a distrustful patient—e.g., because of personal negative experiences with the health care system, emotional instability, or a physician’s bad reputation. Or it may begin with a doubtful physician—e.g., because of a patient’s low compliance as reported by the referring colleague, because of a lack of knowledge about the patient’s disease, or because of a family relationship with the patient that makes the physician uncomfortable. Or both patient and doctor may be having a bad day and thus not inclined to build a new relationship. Examples of reasons for beginning without trust are endless. Trust must be built—often from scratch or in unfavorable conditions—to create that magnificent bond between patient and physician that is called the therapeutic alliance.

Translating the famous postulate according to which when we speak, we do things [49], it is possible to say that when we communicate, we (can) build a relationship based on reciprocal trust. Moving it to the context of doctor-patient interactions, with reference to the communication of severe diagnoses, it is evident that it is even more important to properly communicate to establish a solid therapeutic alliance. Understanding what happens in this always delicate interaction, that it is about sensitive topics (patients themselves, their health status, their lives, and more), is at the core of well managing it. This means not only that the speakers need to get their bearing in the conversations, but also that they are asked to acknowledge the emotionally charging conversation they are sharing. For example, the physician’s words may figuratively wound the patient’s mind ([50], p. 27). A moment later, they may be a balm for the patient’s mind and body, as in the beautiful poem “A fil di voce” ([50], p. 47).

It is essential to understand how language works and see the multiple layers that contribute to shape the dialogue. It is not possible to focus on interactions without having in mind Grice’s work on the implicit dimension of language [51, 52]. On the implicit dimension of language in the context of doctor-patient communication, see also [53]. Especially physicians, who lead the communication [54], must be aware of their role and choose words carefully [55]. Moreover, physicians and patients have different frameworks of reference in terms of meaning, which contributes to make the exchange of information tricky. In Austin’s words [49], it is more difficult than in a daily conversation to satisfy the *felicity* of the interaction.

Physicians must also be mindful of the possible crisis that for patients is entangled in the diagnosis, and questions that may remain unanswered on existential dilemmas. Communicating always means being in contact with the other speaker, in a mutable and (ideally) constantly improving and developing relationship. Since medicine is not based only on quantitative data and medical facts, it needs to embrace patients’ stories and perspectives as well. Thus, a good doctor must be a good communicator both actively, by effectively conveying information to patients, and passively, by listening to them. Being good communicators for physicians also means letting the patients’ stories come to light [56–58]. Physicians and patients must understand each other to collaborate; they must reciprocally ask questions to be sure that they are on the same page; they must make their perspectives explicit and share them. This contributes to strengthen reciprocal trust and the sense of being in safe hands. Patient’s life and death are at stake, especially in the context of a severe diagnosis, and not only the conversation must be effective, but also respectful of the sensitive moment they are going through. Recalling Ofri’s words about conveying a severe diagnosis: “[…] this would be where I’d gently work my way into what is by far the hardest conversation a doctor can have with a patient. This time, though, I started with a question: *How much would you like to know*?”. This is a clear example of the moment when she realized her duty to respect his patient’s desires and decisions; and maybe her empathic attitude well-played in this context: in fact, the patient asked not to be fully informed about his disease and “several weeks later, he died at home, by all accounts peacefully” [59].

### 3.2 Philosophy in action

Philosophy has played a leadership role in the design of the study protocol even from a methodological standpoint. Although we have borrowed established methods from sociology and tools from psychology, philosophy serves as the foundational framework for our data collection and analysis. Given that our objective was to study doctor-patient communication at the time of PDAC diagnosis from a perspective that extends beyond mere technical description, a structural configuration was required that aligned with the profoundly humanistic roots of our research. As the details of the study protocol structure have been comprehensively discussed in [31], I will here summarize the pivotal phases specifically highlighting the contribution of philosophy to each one (see Table 1).

**Table 1:** Phases of the study protocol *COMMUNI*.*CARE* and, for each, the contribution of philosophy.

PHASE	PHILOSOPHICAL QUESTIONS
Doctor-patient interaction about the diagnosis	*How do speakers act in the context of diagnosis*?
PHE-Scale (PHE-s®) collection	*Is patients’ understanding blurred by emotions*?
Semi-structured interview with the patient	*Are patients aware of communicative dynamics*?
Qualitative Content Analysis	*Did misunderstandings occur*?
Statistical Analysis	*Are there correlations between patients’ understanding and other variables*?

#### 3.2.1 Doctor and patient in dialogue

Given the assumption that communication and trust are strictly interrelated, our aim was to study the communication of the diagnosis with the philosophy of language lenses. We know well that the meaning of communication relies in what is said as far as in the unsaid. When it comes to understand difficult information, both in terms of technicality and of psychological weight, it is even more important to understand what happens in the interaction.

The observation of the phenomenon was thus an indispensable starting point to examine the speakers in the delicate moment of information transmission/reception. Observing reality is indeed the cornerstone of applied and rooted in the real-world philosophy that aims at describing problems and, ideally, proposing solutions.

The moment when the physician conveys the diagnosis to the patient cannot be merely reduced to an act of description: instead, it entails a chance for the patient (and their family) who receives it, thus directly modifying their reality. It is evident that put in this way, the communication of diagnosis is itself a perfomative act [49]. As previously mentioned, the concept of *felicity* and *infelicity* in Austin refers to a linguistic act that is or is not successful. How is this act performed? How do speakers re-act in this context? In Austin’s words, are they *happy* with the interaction?

#### 3.2.2 Mixing methods

As already stated, the protocol here described is strongly interdisciplinary: this means that philosophy collaborates with other disciplines in a joint effort to achieve the most accurate possible overview of the research topic. Doing this implies to include in the methodology tools coming from other disciplines. Among these, the Patient Health Engagement Scale (PHE-s® [60]) is a psychological validated scale that aims to detect patients’ level of engagement in their relationship with the physician.

The PHE-s® was administered to patients at the end of the diagnostic visit to assess patient’s emotional status. It determines the patient’s self-perception, starting from a feeling of overwhelm caused by the illness and progressing to a fully engaged individual in the treatment journey. Since it comprises of five questions with seven-choice answers, it is non-invasive and can be filled quickly: it was the right tool for us in order not to overstimulate the patient.

The philosophical question behind the choice of using this scale was: are emotions blurring patient’s understanding? It is not difficult to imagine that in such delicate moment feelings are at stake; but it was a main point for us not to generalize starting from a top-down approach, instead being able to study case by case, patient by patient. Thus, the questions that guided our choice of introducing this psychological scale were: is this individual in black out, thus with a distorted comprehension? Is there a real correlation between the vigour of emotions and effective communication? Do patients’ feelings impact the information they receive and the way they receive them?

#### 3.2.3 Patients’ voices

The next step after the observation of the visit could only be to give voice to those who most directly assume the role of subjects in the interaction: the patients themselves. The interviews were conducted with them in the context of a deafening diagnosis, striving to articulate their beliefs and experiences.

The post-diagnosis interviews represented a central passage in the present research. As interviewer, since the non-participant observation was precluded by the emergency situation, I listened to the physician-patient interaction before conducting the interview to collect possible interesting events happened during their conversation. The primary goal of my interviews was to granting patients the opportunity to express their point of view on the conversation they had with physicians and, broadly, on their opinion about the importance of communication in building a therapeutic alliance with their doctor. A semi-structured interview was chosen as the best way to reconcile the possibility for them to explicit their thoughts and the need to explore specific topics. These were: their view about communication in therapeutic relationships; about a possible link between communication and trust; any hurdles they may have experienced in their conversation with the doctor; and the role of their companions/caregivers in the conversation. Due to the dynamic nature of the tool of semi-structured interviews, it was possible to adapt questions and facilitate the organic progression of the interview framework.

Interviews with patients had a double purpose. Firstly, we went through patients’ words to find inspiration and important topics to guide our analysis. For example, if a patient highlighted the importance of raising questions when receiving a diagnosis, had s/he been able to ask for clarification during the interaction with the physician? Because of the extremely delicate topic, it happened that patients showed themselves upset and terrified. Did the doctor pay attention to the small questions that revealed their emotional status during their interaction?

Since philosophy of language tools generally provide a meta-analysis of communication, our purpose was to complete this analysis introducing our understanding of patient’s awareness of the communicative dynamics. Exploring their active knowledge of the experienced dynamics helped us in distinguishing what happened actively and what passively; paraphrasing Austin’s words, what they *did* consciously and what spontaneously. Thus, we checked patient’s awareness of occurred misunderstandings (or their willingness to talk about it) and later on we crossed it with our labeling of their interactions with physicians.

All the analysis of the interviews were performed using phenomenological analysis to be sure to keep the focus on patients/speakers’ experience.

#### 3.2.4 Qualitative content analysis

After all the transcriptions of the interviews between patients/their caregivers and physicians were completed, we performed the qualitative content analysis. The latter represents a systematic approach that is appreciated for its ease of use, allowing for data and result interpretation and reinterpretation.

It was conducted by two philosophers. After uploading all the doctor-patient interactions on MaxQda [61], a software specifically developed for this type of analysis, we created our two sets of labels. The first one was intended to identify the kind of misunderstandings that happened during communication; it was based on [62]. This codebook provides a set of labels, that we integrated with a second set of three labels aiming to identify the moment when the misunderstandings occurred (Anamnesis, Diagnosis, and Treatment). Since we wanted the labeling to be as objective as possible, we did a joint training on seven documents before performing the analysis independently. We then verify our agreement and considered it satisfying (agreement 97.9%; Cohen’s Kappa: 0.98 [63]). In Table 2, labels are explained highlighting their contribution in better understanding the problematic passages of the analyzed conversations.

**Table 2:** Description of felicity/infelicity applied to the codebook; examples from our data (table modified from [32]).

Label	Description	Example	Felicity/Infelicity
*Clarification*	The hearer asks the speaker to better specify the meaning of their utterance, as some of its components can have different interpretations. No interpretative hypothesis is advanced; only a question is asked concerning a component of the speaker’s utterance.	Doctor: […] the histology report is positive for “ductal adenocarcinoma”. Patient: *Eh*, *what does that word mean*?	Infelicity then solved: one speaker does not understand the meaning of the other’s words. Due to the speaker’s question, the misunderstanding is solved.
*Check for Understanding*	The hearer expresses their doubt, as they are uncertain whether they have understood correctly what the speaker said.	Doctor: Yes, [to check] the port with cath. Patient: *Ok*, *so*, *I have to come here to check the port with cath*. Doctor: Yes.	Felicity: one speaker asks for a verification of their understanding, that is ultimately correct.
*Declaration of Lack of Understanding*	The hearer acknowledges meta-dialogically that they cannot understand, or that the interpretation that they have achieved is not acceptable.	Doctor: Ok, so, on March 22 we have this blood exam with CA19-9 that corresponds to 69. Patient: *Excuse me*, *what does it mean*?	Infelicity then solved: one speaker openly states that does not understand the other. The open statement allows the other speaker to better explain.
*Semantic Alternative Understanding*	The hearer interprets the speaker’s turn by specifying its meaning in a way that is not acceptable or accepted. The speaker corrects the hearer’s alternative interpretation of the semantic representation of his/her utterance. The semantic representation refers to the phenomena referred to as “explicatures” in addition to syntactic or semantic disambiguation.	Doctor: I will prescribe some ordinary pills to take when you eat to better absorb the nutrients. Patient: *You already gave me some stuff to go to the bathroom…* Doctor: That was because you were a little constipated, madam.	Infelicity: one speaker refers to an alternative set of meanings that does not correspond to the other’s one. The latter does not make this explicit and the misunderstanding remains at stake.
*Pragmatic Alternative Understanding*	The hearer interprets the speaker’s turn by drawing inferences that are not acceptable or accepted. The speaker corrects the hearer’s alternative interpretation of the intended purpose of speaker’s utterance. The intended purpose includes implicatures, presuppositions, or in general pragmatic inferences drawn from an utterance.	Patient: Thank you. *Ok*, *so if we get positive results in six months you will try to operate*? Doctor: No, madam, I never said anything about a surgery, no.	Infelicity: one speaker infers wrong consequences from the other’s words. The latter does not make this explicit and the misunderstanding remains at stake.
*Irrelevance*	The hearer continues the conversation with a turn that is incoherent either pragmatically (e.g., request of information followed by an acknowledgment) or for topic (change of subject) with the previous turn.	Doctor: Ok, so, for sure today I’ll ask you about blood exams, I’ll ask you about some stuff that it will be important to do. Patient: *I ate a little*. Doctor: No, no, you won’t do it now.	Infelicity: one speaker answers with different meanings than the ones at stake in the conversation.
*No Uptake*	The hearer fails to take into account the other’s turn by interrupting the dialogue (silence) or continuing the dialogue without considering the interlocutor’s turn.	Patient: And then I have this ache right in the pit of my stomach, as soon as I finish eating…sometimes it’s very serious, I have really bad cramps… [Doctor is compiling the medical records] Doctor: *So*, in this space I write *P’s surname*, *correct*?	Infelicity: one of the speakers does not reply to the other’s words.

We selected this codebook as it is based on linguistic evidence, as openly stated by its authors (in fact, they refer to the codebook as LEPU, the acronym for Linguistic Evidence of Problematic Understanding). Since our philosophical approach has its roots in the belief that language and action are intertwined and, going further, that language is itself and act, we find this codebook aligned with our vision of doctor-patient interaction. In fact, the felicity of the linguistic act is not met when a misunderstanding occurs; the latter could be solved or not, thus succeeding in communication or still pursuing the speakers’ mismatch of meanings.

As it emerged from the table, further investigations should delve into the classification of misunderstandings to better highlight the most dangerous passages, i.e., where infelicity happens without being properly solved.

#### 3.2.5 Statistical analysis

Pertinent data were collected during visits and interviews using an anonymized Case Report Form (CRF). These recorded variables encompassed patient age, gender, region of origin, educational level, histological PDAC diagnosis date, disease stage, and proposed treatment plan. Patients were retrospectively categorized as compliant or non-compliant based on adherence to the proposed treatment plan (despite being aware of the on-going debate about the use of the expressions *compliance*, *adherence to treatment*, and *concordance* [64–66], in this context, we decided to use them as synonyms). Additionally, patients’ overall survival at the study’s conclusion was documented.

To investigate the influence of patient comprehension on engagement and compliance, logistic regression analyses were conducted, using high PHEs® level and compliance as outcome variables, and incorporating patients’ characteristics, disease features, and degree of communication as explanatory variables. All the statistical calculations were performed by clinicians.

It is worthy to notice that when we read our data, we extracted quantitative results, but always based on philosophical reasoning. We obtained numerical outcomes, such as how many misunderstandings occurred, which ones were the most frequent, and the percentage of time spoken by physicians and patients. Nonetheless, all these numbers were based on qualitative questions: when do patients’ doubts and fears find space to be expressed during the visit? In which form? Is there a difference in terms of attitude between patients with a more advanced tumor and others? The numerical results we calculated have philosophical roots that allowed us to interrogate the software in a precise direction. Where the outcomes have been translated into numbers and percentages, the philosophical matrix of the research has enabled a nuanced understanding and prevented them from being reduced to mere descriptive data of an interaction that must always be represented in its utmost delicacy.

Conducting and interpreting the quantitative analysis in this way allowed us to consider possible areas and methods of future interventions to improve the effectiveness of doctor-patient interactions (and, more generally, healthcare professionals and patients/families), thus fostering their reciprocal trust and giving strength to their therapeutic alliance.

### 3.3 Philosophy in outcomes

The present article is not conducive for discussing the results of the study in a thorough manner (that can be read in [32]). Nonetheless, a glimpse at some previous outcomes we obtained may be useful to grasp the potential of interdisciplinary research. While describing present issues, the philosophical approach can offer possible solutions and open research questions. In this specific case, we searched for moments of dialogic difficulty between the doctor and the patient, identified them, and described them. This allows for the subsequent formulation of potential "adjustments" to those moments of difficulty based on observations and problematization. As mentioned above, philosophy is also directed towards the future: finding results rarely fails to pose further questions, suggesting new possible research directions. Among the prerogatives of philosophy is undoubtedly continuous study and an evolving understanding of reality, which itself is in a perpetual state of transformation.

#### 3.3.1 A brief overview of interesting results

To adhere to ethical guidelines, informed consent forms containing the term "adenocarcinoma" (cancer) were presented and signed at the visit’s conclusion, preventing an untimely disclosure of the diagnosis. Nonetheless, it is interesting to notice that, despite dealing with diagnostic interactions, physicians had the tendency not to directly name the diagnosis to patients. Terms as adenocarcinoma, cancer, and tumor are cited when speaking with patients about their conditions and suggested treatments, but none of the involved doctors ever said, “This is your diagnosis/You have been diagnosed with…”. There are many guidelines that aim to support clinicians in communicating bad diagnosis in the best possible way; among these, quite famous is the protocol SPIKES, originally thought for patients with cancer [67, 68] and then applied to other context (i.e., revised for nurses: [69]; to deliver the diagnosis of dementia: [70]). Could this be a way to introduce patients to their condition without being traumatized? Is this a paternalistic approach that does not fully disclose what is happening to patients? From a philosophy of language perspective, it is notable that one of the main subjects of the speech is rarely nominated, contributing to shape an environment of discussion that orbits around a mysterious disease, present but not nominated, detectable but invisible.

Despite the seemingly balanced participation in the conversation between the two parties involved, physicians talked much more than patients and their companions during the diagnostic visits. It is expected in this sensitive context, since physicians have a lot of information to convey to patients [71, 72]; however, it could also be a hint that something not yet investigated is happening here [73, 74]. Further explorations may delve into new research questions that arise from the above results, such as: is there enough space for patients’ questions in terms of time? Does patients’ attitude tend to be focused on listening instead of speaking/asking? Do physicians help patients in elaborating the news they just received and feeling free to express their thoughts about it?

Albeit patients did not speak much in these conversations, they used to ask questions, especially concerning the suggested treatment. Patients in this context showed themselves to be almost exclusively interested in proper understand what they are supposed to do now, at the very present moment: which exams they must perform, how to obtain the exemption for various prescriptions, how long the treatment would last… Caregivers are aligned with them, asking practical details, such as if they are recommended to pick up patients after the chemotherapy, if they must inform the General Practitioner about the disease, and so on. It is a practical approach that may have different reasons: the need to focus on what it is possible to do, meaning how to be active in the context of a severe disease; or also a removal process not to fully realize the importance of the diagnosis itself. Of course, it is not easy to point out the reason behind this behavior, which can be seen as either positive or problematic.

It is also a significant result that during the interviews all but two patients referred no issues in their interaction with physicians. One patient described his difficulties in asking questions as the physician did not give him the space to do it and was visibly annoyed when interrupted. The other patient was yet emotionally affected by technical terms used by two physicians when discussing his tumor among themselves but in front of him; even though they then spent some time to explain the meaning of what they were pointing out between them, he was still significantly impacted by what he considered lack of empathy. Why did patients not acknowledge the issues we detected in their conversations with physicians? There can be many reasons and of course we still do not have the answer: was the reason they removed what went wrong because they were, all in all, satisfied? Or maybe because they preferred to focus on positivity in such a sensitive moment for them? It is also possible that, although the questions’ aim was to address any possible kind of hurdles they experienced in communication, they perceived the interview as a self-assessment test and preferred to confirm that everything went well. In this direction, literature suggests that people in general, and patients in particular, tend to *save face* in a context in which not only they may be judged as incompetent, but also are vulnerable subjects [75, 76].

The results of this interdisciplinary study conducted with a strong philosophical perspective inherently ensure adherence to the complex reality of the problem, ranging from the human component to the more technical aspects. In this context, the results are not merely derived from a technical analysis of language mechanisms but also from a constant focus on the profound experience that is the diagnosis of PDAC. Furthermore, the interpretation of the results maintains a philosophical perspective, allowing for an expansive examination of the data that does not fragment the investigation but preserves the complexity of the observed phenomenon.

As expected, the study pilot has raised questions that merit further investigation in the future. It suggests possible directions to enhance reciprocal trust by means of communication. As proposed at the very beginning of this article, a valuable outcome would possibly be the drafting of guidelines for good communication in the context of severe diagnosis. Benefitting from the present study, these guidelines would be strongly rooted in the linguistic dynamics of language with the forward-looking understanding of its connection with emotions and its impact on patients’ compliance.

## 4. Additional considerations

There is no sense in philosophy without exchange. At the very core of this field, there is the assumption that dialogue is fruitful and enriching. In the context of this study protocol, philosophical dialogue can be identified in multiple directions. Firstly, the effort to pursue an open-minded attitude comes within philosophy, that is itself dialogical. Multiple kinds of philosophy are at stake, starting from philosophy of language and philosophy of science, passing through the inclusive perspective of philosophy of medicine, and reaching the challenging ethical deliberations. Then, the dialogue is nurtured with other disciplines involved (medicine, psychology, and sociology) to build a research project responding to real needs, based on real facts, and seeking for constant improvement.

The paramount philosophical dialogue of this study protocol is the one with human beings. It unfolded progressively as the different phases of the research were completed, from the teamwork with colleagues to the dissemination for the public. The most demanding yet inspirational part was the interaction with patients. It was inevitable to touch upon topics unrelated to the research’s scope, but undoubtedly of huge importance for them. “May I ask you something? If I undergo chemotherapy, will I lose my hair?” “It’s just that I’m very calm […] I like to stay positive, never negative, that’s the most important thing. What do you think?” “Why did this illness come to me?” These are some of the most impacting questions I experienced during the interviews with patients; some of them were crying, others holding hands with their loved ones (“There’s my husband here with me…”). I did not have answers for them; all I could do was to suggest talking with clinicians and dedicated psychologists, once again confirming the interdisciplinarity of the real world itself. It is often hard to grasp the meaning of what happens in one’s life. When speaking with patients, people with their own stories and fears and families, I learnt myself to bring back to simplicity the background noise of existential questions I could not answer. Thanks to one of them who said, “Just…thank you. Having chatted like this is already a small release that helps me a bit”, I knew the potential benefits of just being able to speak with someone about their experience. Confirming the importance of sharing thoughts and experiences, I was myself grateful for their give-and-take.

## 5. Discussion

The interdisciplinary approach of the study protocol Communi.CARE has pros and cons, that I see as intertwined and have their roots in the struggles experienced in creating a dialogue between different fields: this is unusual and not yet consolidated. When designing the protocol, we had to pay attention to being on the same page and less possible invasive for each other. For example, physicians need to preserve their rhythm and be able to include patients in the study when it was convenient for them; philosophers required a training with clinicians about the disease but paying attention not to be biased in the interviews with patients. It was sometimes difficult to understand each other and prevent misunderstandings among the members of the group. Not every member was familiar with the methods we employed, and we had to explain to each other the path of data collection and analysis. The final phase of data crossing was particularly challenging because it required a deep discussion to interpret reciprocal results.

The continuing dialogue was useful both to solve the above-mentioned issues and for reciprocal professional growth. It allowed all the experts involved to enrich each other with new competences borrowed from colleagues. I would say that we went even beyond this, with a step in the direction of what Choi and Pak [33] call *transdisciplinarity*, a cake “in which the ingredients are no longer distinguishable, and the final product is of a different kind from the initial ingredients” (p. 359). Philosophy was indeed the leading perspective, always re-conducting numbers and impersonal results to the intrinsic complexity of human beings. Of course, there is still a long way to go to hopefully be able one day to point out a research protocol and say, “This is a well-done, methodological strong, and with useful results transdisciplinary study!”. With ours, we tried to do our best to design a fruitful interdisciplinary approach that aimed to give interesting results for the medical world and raise questions for further reflection. Anticipatively, upcoming studies would delve into the issues emerged in this research with larger samples.

Despite the continued challenges and substantial resource investments (both personal/professional and in terms of time and finances), fostering dialogue among diverse professionals proved to be highly beneficial. In the context of doctor-patient interaction, I strongly believe that it would endorse effective and ethical communication in critical life junctures. The intertwining of effectiveness and ethics is undeniable when dealing with individuals: one forms the foundation of the other. Effectively conveying information is impossible without respecting the patient who receives life-altering news. Similarly, providing obscure information in a convoluted manner displays a lack of respect for patients. In a broader sense, interdisciplinary frameworks would drive investigations grounded in real-world necessities, rather than detached from its human side.

In my opinion, the interdisciplinary approach is incomplete without philosophy among the disciplines in dialogue. Instead, as I suggested throughout the article, philosophy may be the right discipline to guide the structure of an interdisciplinary project. The main reason dwells in its human roots: the complexity of human nature cannot be forgot when philosophy leads the way, even though the path aim to be scientific. This is a guarantee of a research that considers the human beings involved not as mere numbers, but as subjects. The intrinsic dialogical nature of philosophy allows to put in dialogue different fields of knowledge and to reach a holistic perspective, in which data and results are read keeping into account their many facets.

Interdisciplinary is not new; nonetheless, since it is not that common to use it, it will be fascinating to see the flourishing of new methods and studies that are structured around it. In times when one health is encouraged and advantageous [77], this would be indicative of a society that do cares of its citizens, whether they are healthcare professionals or patients.
